# A study protocol for exploring and implementing a surgical pharmaceutical service model in drug treatment management for patients with osteoporosis fracture in China

**DOI:** 10.3389/fmed.2025.1502360

**Published:** 2025-03-12

**Authors:** Jieluan Lu, Yi Luo, De Cai, Yali Wang

**Affiliations:** Department of Clinical Pharmacy, First Affiliated Hospital of Shantou University Medical College, Shantou, China

**Keywords:** osteoporosis, osteoporotic fracture, pharmacists, fracture risk assessment, adherence

## Abstract

**Background:**

Osteoporotic fractures are serious consequences of osteoporosis, which is a condition that can be prevented through effective therapeutic strategies, including the use of anti-osteoporotic medications. However, a significant treatment gap exists in elderly patients with osteoporotic fractures. A multicenter study conducted in China reported that only 20% of elderly patients with hip fractures received appropriate pharmacotherapy post-fracture. This lack of treatment resulted in an increased risk of refracture associated with osteoporosis. Pharmacist-led interventions have proven essential in medication management for osteoporosis and related fractures, potentially bridging the treatment gap. Accordingly, a protocol was developed to assess the impact of pharmacist-led interventions on increasing the continuation rates of anti-osteoporotic drugs and reducing the risk of refracture in patients with osteoporotic fracture, compared to no interventions (grant number: YCTJ-2023-15).

**Methods and analysis:**

This study is a single-center, prospective, and randomized controlled trial. The targeted participants in this protocol were patients aged above 50 years who had been diagnosed with osteoporotic fractures in China. Eligible participants were randomized into intervention and control groups in a 1:1 ratio using a dynamic stratified block randomization method. The control group received standard care, and the intervention group received standard care combined with pharmacist-led care. The intervention group received comprehensive pharmacist-led interventions, including pharmaceutical ward rounds and medication reconciliation, refracture risk evaluation, recommendations to physicians, patient education, and counseling. A 2-year follow-up was conducted to evaluate the outcomes between groups through telephone interviews, pharmaceutical clinics, and e-hospital pharmacy practice. The primary outcome is the ongoing treatment rates of anti-osteoporotic drugs. The treatment rates are defined as the ratio of patients who remain on anti-osteoporotic medications at each follow-up visit to the total number of enrolled participants. Secondary outcomes include treatment initiation rates, medication adherence, re-fractures, and use of drugs that increase fall risk, the frequency of bone mineral density (BMD) assessments, the incidence of inappropriate medication use, adverse drug reactions (ADRs), and patient satisfaction with osteoporotic fracture treatment. Refracture rates were evaluated through a 2-year follow-up, while BMD were measured at baseline, 1 year, and 2 years using dual-energy X-ray absorptiometry (DXA). ADRs and the inappropriate use of medication were monitored through self-reports and medication reconciliation. Patient satisfaction were assessed using the Treatment Satisfaction Questionnaire for Medication version II (TSQM-II). Ethical approval was obtained from the Committee of Ethics of the First Affiliated Hospital of Shantou University Medical College (approval number: B-2023-194). The statistical analysis was performed using Statistics Package for the Social Science (SPSS), version 23.0.

**Discussion:**

We hypothesize that analyzing pharmacists-led interventions provide valuable insights into how pharmacists improve treatment outcomes for patients with osteoporotic fractures. This study aims to address the existing knowledge gap regarding the effectiveness of pharmacist-led interventions in improving the management of osteoporotic fractures in China.

## 1 Introduction

Osteoporosis (OP) is a systemic disease characterized by reduced bone mass and strength, primarily affecting the elderly population. In China, osteoporosis affects 32% of individuals over the age of 65 years, with women comprising 51.6% of this group (1, 2). Osteoporotic fractures (OFs), also known as fragility fractures, are a prevalent and serious consequence of osteoporosis, significantly affecting the quality of life and elevating the economic burden on both patients and society. The prevalence of OFs in China is predicted to reach 5.99 million by 2050 (3), highlighting the urgent need for effective management strategies.

Effective treatment for osteoporosis, including the use of anti-osteoporotic medications, has been shown to reduce the risk of subsequent fractures by 14–22% (4) and mortality by 19–36% (5) in patients with hip fractures. However, a significant treatment gap persists, particularly among elderly patients with osteoporotic fractures (6). A cohort study has revealed that only 27.7% of women received osteoporosis medications within 12 months after an index fracture, while 72.2% remained untreated (7). A multicenter study in China reported that only 20% of elderly patients with hip fractures received appropriate pharmacotherapy after their fractures, while over 80% remained untreated (8). This significant treatment gap is further worsened by low treatment initiation and adherence rates, which are critical factors in osteoporosis management (9). The risk of drug-associated issues in elderly patients with osteoporosis is aggravated due to multiple prevalent diseases, polypharmacy, and surgical approaches. Elderly patients with osteoporosis are at a higher risk of falls and refractures (10). Polypharmacy may increase this risk of falls and refractures due to the presence of drugs that increase fall risk (11), resulting in the treatment failure for osteoporosis. Therefore, further medication management of polypharmacy is needed for elderly patients with osteoporotic fractures.

Pharmacist-led interventions have been shown to play a crucial role in addressing treatment gaps in osteoporosis (12). A previous study involving 108 patients with hip fractures revealed that pharmacist interventions improved the quality of medication treatment, reduced the use of potentially inappropriate medications, and optimized pharmacotherapy at the time of discharge (13). Pharmacist-driven osteoporosis management has resulted in an increase in the percentage of rural veteran patients who underwent dual-energy X-ray absorptiometry (DXA) screening (14). Overall, pharmacist interventions significantly improved the compliance rates with guidelines for glucocorticoid-included osteoporosis compared to patients who did not receive the intervention (15). A study comparing the impact of pharmacist-led and nurse-led interventions in postmenopausal women with fractures demonstrated a significantly higher increase in the initiation rate of osteoporosis drugs driven by pharmacists compared to nurse-led interventions (16). Only a few studies have investigated the relationship between pharmacist interventions and osteoporotic medication adherence (17–19). However, the impact of pharmacist-led interventions on osteoporosis management, particularly in the context of fracture prevention and treatment adherence, remains underexplored in China. The effectiveness of pharmacist interventions in conventional therapy is still undefined, despite several studies demonstrating a positive correlation between pharmaceutical interventions and osteoporotic treatment.

Given the significant treatment gap and the potential benefits of pharmacist-led interventions, this study aims to evaluate the effectiveness of a comprehensive pharmacist-led intervention model in improving treatment rates of anti-osteoporotic drugs among elderly patients with osteoporotic fractures. This study provides valuable insights into the role of pharmacists in osteoporosis management and contribute to the development of effective strategies for improving patient outcomes in China.

## 2 Materials and methods

### 2.1 Study design

This protocol follows a single-center, prospective, randomized, and controlled design, in accordance with the Standard Protocol Items: Recommendations for Interventional Trial (SPIRIT) 2013 Statement. The recruitment date was from 1 October 2023 to 30 June 2024. Patient enrollment for the study is ongoing.

### 2.2 Participants

Eligible participants were recruited from The First Affiliated Hospital of Shantou University Medical College. The National Osteoporosis Foundation guidelines recommend that medication consideration be given after hip or vertebral fractures in postmenopausal women and men aged 50 years and older (8). The study focused exclusively on patients aged 50 years and older who have been newly diagnosed with osteoporotic fractures, including hip, vertebral, and wrist fractures, according to the International Classification of Diseases, Tenth Revision, Clinical Modification (ICD-10-CM) (20). Therefore, the inclusion criteria are as follows: (1) patients aged 50 and older; (2) those newly diagnosed with an osteoporotic fracture, including hip, vertebral, and wrist fractures.

Exclusion criteria are as follows: (1) Cognitive deficit (cognitive ability to respond and perform the exercises assessed by the Mini-Mental State Examination; MMSE); (2) severe liver insufficiency (Child-Pugh lever C) and severe renal insufficiency (eGFR <30 ml/min/1.73m^2^); (3) use of glucocorticoid drugs within the past 6 months; (4) presence of a malignant tumor or an expected life expectancy of less than 2 years; (5) hospitalization duration of less than 48 h; (6) lack of basic information; (7) patients who died during hospitalization; and (8) unwillingness to participate in this study or refusal to sign the informed consent form.

### 2.3 Withdrawal criteria

Patients can voluntarily withdraw from this clinical trial at any time. Additionally, patients were required to withdraw from the trial if they experience any of the following conditions: (1) Serious adverse events (AEs) occurring at any point during the entire trial process; (2) Inability to continue participating in clinical examinations and follow-up due to unexpected reasons.

### 2.4 Randomization and allocation

The patients diagnosed by orthopedists were screened for eligibility based on clinical inclusion criteria. The medical records of those who met the criteria were then forwarded to surgical pharmacists. Patients were randomly assigned to standard care or pharmacist-involved care (1:1 ratio) by team members not involved in clinical pharmacy services, based on whether they received intervention from surgical pharmacists. A dynamic stratified block group randomization method was used to randomly group participants, stratified by sex and age of participants, using R package blockrand (R version 4.0.3).

### 2.5 Standard care

Orthopedists conducted routine evaluations and therapy for all enrolled patients with OF. This process included diagnosing the condition, conducting ward rounds, prescribing medications, performing surgical interventions when necessary, and documenting medical records. Moreover, the orthopedists provided a brief overview of osteoporosis and key instructions to patients with OF. However, surgical pharmacists were not included in discussions related to osteoporosis during this process.

### 2.6 Surgical pharmacist intervention pathway

The goals of pharmaceutical care involved both orthopedists and patients with osteoporotic fractures. On the one hand, surgical pharmacists provided prescription reviews and consultations with physicians on treatment options for osteoporosis. On the other hand, pharmaceutical care for patients consisted of explaining the importance of diagnosing and treating osteoporosis, providing guidance on the fall risks, and outlining the main precaution related to the use of anti-osteoporotic drugs.

Patients allocated to the intervention group received pharmacist-involved care to identify the effects of pharmaceutical services in OF treatment. On the basis of the standard care, surgical pharmacists, dressed in standard and appropriate professional attire, conducted bedside pharmaceutical consultations individually at three distinct stages: on the first day of admission; within 1 week post-surgery or once the condition is stable; and on the day of discharge. This interaction typically spans a duration of 10–20 min.

#### 2.6.1 Day 1 of admission

A drug reorganization form will be completed to collect medication history, drug allergies, and current medication use, as recorded by the surgical pharmacist through pharmaceutical diagnosis. The bone mineral density (BMD) measurement and exact therapeutic plans are formulated by orthopedists. Consultative reviews are further established by surgical pharmacists, focusing on: (1) the usage and dosage of therapeutic drugs; (2) soluble media; (3) drug contraindication; (4) risks of adverse drug reactions (ADRs); and (5) collaborative drug treatment for chronic disease, including diabetes, hypertension, and hyperlipidemia. Surgical pharmacists will send a new medicine/deprescribe or a change based on the consultative review to orthopedists if indicated. Otherwise, it is necessary for surgical pharmacists to communicate with orthopedists and nurses for medication errors, adjustment in current medicines, and provide medical consultation.

#### 2.6.2 Postoperative or stable condition within 1 week

First, the surgical pharmacist uses the Fracture Risk Assessment Tool (FRAX) to evaluate the risk of fracture. Second, the individualized therapeutic options for osteoporosis are formulated under the guidance of the Clinical Pathway of anti-osteoporotic drugs of patients with osteoporosis formulated by surgical pharmacists and orthopedists. Third, all participants will be educated by surgical pharmacists, including the usage of anti-osteoporotic drugs, the importance of persisting with osteoporosis medication, maintaining physical activity, and prevention of falling. Furthermore, patients will be provided a brochure on preventing fractures to enhance the consciousness of osteoporotic fractures.

#### 2.6.3 One day of discharge

The current drug list, along with guidance for self-care, will be conducted. All participants will receive an educational brochure explaining the customized treatment of osteoporotic fracture, such as dosage, precautions for medicine taking, ADRs monitoring, and specific follow-up time.

#### 2.6.4 After discharge

We evaluate outcomes of pharmacology interventions during 2 years following the start of discharges, using telephone interviews, clinic visits, and pharmacy practice in e-hospitals. Follow-up time points are set at 1 week, 1 month, 3 months, and then every 3 months thereafter until 24 months post-discharge.

### 2.7 Regular follow-up and engagement

Participants who did not take the medication as scheduled beyond 8 weeks of the expected follow-up period, as well as those who no longer wish to participate in the study, were discontinued from the study.

To address the potential for missing data due to the high dropout rate in the elderly population, we will incorporate an intention-to-treat (ITT) analysis approach. ITT analysis will include all randomized participants in the analysis according to their assigned group, regardless of whether they completed the follow-up or not, which may minimize the bias introduced by missing data and provide a conservative estimate of the treatment of the treatment effect (6).

Participants will be followed up at 1 week, 1 month, 3 months, and then every 3 months thereafter for a total of 24 months. This frequent and structured follow-up schedule is designed to enhance participant retention and ensure comprehensive data collection.

Multiple follow-up methods, including telephone interviews, pharmaceutical clinic visits, and pharmacy practice in e-hospitals, will be used to accommodate different preferences and accessibility of elderly patients.

Trained pharmacists will conduct telephone interviews to gather detailed information on medication use, adverse reactions, falls, and fracture events. Face-to-face consultations at the pharmaceutical clinic will provide personalized medication education and counseling, addressing patient questions and offering the necessary support. For patients unable to visit the clinic, real-time pharmacy practice in e-hospitals via video conferencing or instant messaging will ensure timely information and support.

To ensure comprehensive coverage and maintain data quality, all research staff involved in data collection, including pharmacists and clinic personnel, underwent comprehensive training on the study protocol, data collection methods, and the use of standardized tools. Additionally, we involved family members or caregivers in the follow-up process to provide accurate information on participants’ conditions and medication adherence, ensuring complete and reliable data.

To encourage participant retention, educational materials and personalized support were provided during each follow-up session.

### 2.8 Outcome assessments

#### 2.8.1 Primary results

The primary outcome, assessed within 2 years post-discharge, is defined as the treatment rates of anti-osteoporotic drugs among patients receiving pharmacist-led interventions compared to those on conventional treatment. The treatment rates are calculated as the ratio of patients who still use anti-osteoporotic drugs to the total number of enrolled participants.

Ongoing treatment is defined as the uninterrupted use of anti-osteoporotic medications, where any treatment interruption lasting more than 60 days is considered as discontinuation (21). According to previous studies, interruptions in osteoporosis treatment exceeding this duration are associated with an increased fracture risk and reduced treatment efficacy (7, 22).

#### 2.8.2 Secondary results

The secondary outcomes include the rate of initial therapy of anti-osteoporotic drugs, treatment adherence, the rate of falls, the use of medications that increase fall risk, the frequency of BMD assessments, the persistence rate associated with anti-osteoporotic drugs, the incidence of refracture after 1 and 2 years, the incidence of potentially inappropriate medications and ADRs, and patient satisfaction with anti-osteoporotic drugs treatment.

Adherence to anti-osteoporotic drugs is calculated from the beginning of treatment, consisting of 30 days. The medication possession ratio (MPR) will be used to identify the treatment adherence, which is categorized into good adherence (MPR ≥ 80%), medium adherence (50% ≤ MP < 80%), and low adherence (MPR < 850%) (23). The MPR will be calculated as the ratio of days covered by dispensed anti-osteoporotic medications to the total days in the study period.

The proportion of patients using fall-risk-increasing drugs including benzodiazepines, chlorpheniramine, amitriptyline, doxepin, doxazosin, terazosin, zolpidem, eszopiclone, zopiclone, diazepam, lorazepam, Olanzapine, Quetiapine, omeprazole, lansoprazole, warfarin, and rivaroxaban (24) were monitored through medication reconciliations and electronic health records.

BMD was assessed using DXA scans at baseline, 1 year, and 2 years post-intervention. Changes in BMD were measured by comparing T-scores and Z-scores at these time points. To ensure consistency and accuracy, all DXA scans and BMD measurements were performed and verified by certified technicians using standardized protocols and calibrated equipment. Due to the variability in BMD measurements and the potential effects of interventions on BMD, we conducted subgroup analyses to further validate the sample size requirement for these outcomes.

Refracture was defined as any new fracture event documented in the medical records or reported by the patient during follow-up visits. The incidence of subsequent fractures in patients with osteoporosis was typically assessed by monitoring patients over a defined period following the initial fracture, often within 1–2 years, to identify high-risk individuals for targeted interventions (25). Therefore, we further assessed the refracture rate through a 2-year follow-up.

ADRs documented in the Case Report Form (CRF) were systematically monitored and recorded through self-reporting during follow-up interviews, clinic visits, and the review of electronic health records. Additionally, pharmacists conducted regular medication reconciliations to identify potential ADRs and provided management recommendations (26).

The Treatment Satisfaction Questionnaire for Medication version II (TSQM-II) was administered to assess patient’s treatment satisfaction (22). This validated tool assesses patient satisfaction across various areas, including effectiveness, side effects, and convenience.

### 2.9 Data collection and management

The baseline information was gathered, including (1) basic information: age, sex, body mass index (BMI), smoking history, alcohol intake, medical insurance; (2) disease history; (3) primary diagnosis at hospitalization, fracture sites, results of BMD assessments, and anti-osteoporotic drugs; (4) laboratory results: serum creatinine, hemoglobinosinase, cottage transaminase, serum albumin, C-terminal Telopeptide of Type I Collagen (CTX), N-terminal Propeptide of Type I Collagen (P1NP), BMD; (5) surgery-related information: type of surgery, surgical incision, surgical operation category, anesthesia method, American Anesthesiologist Association (ASA) score, surgical level, surgical duration, incision healing category; and (6) other drugs used during hospitalization.

During the intervention, dynamic data collection focused on key aspects as follows: the anti-osteoporosis drug regimen and other long-term medications used by patients prior to admission, any drug allergies, the outcomes of medication reconciliations and physician recommendations, treatment satisfaction assessed via TSQM-II, ADRs, and the incidence of new fractures during follow-up.

To ensure comprehensive coverage and maintain data quality, all research staff involved in data collection, including pharmacists and clinic personnel, will undergo comprehensive training on the study protocol, data collection methods, and the use of standardized tools. Regular communication with participants through multiple channels (telephone, email, and clinic visits) will be maintained to ensure thorough engagement (18). Participants will also receive personalized support, along with flexible follow-up options and reminders to ensure they remain engaged in the study. Information obtained through telephone interviews will be compared with data from clinic visits and pharmacy practices in e-hospitals to identify any discrepancies.

### 2.10 Sample size

The sample size estimation was determined by measuring a clinically significant difference in the primary outcome—continued treatment rates of anti-osteoporotic drugs between the intervention and control groups. To achieve a statistical power of 80% (β = 0.20) with a significance level of α = 0.05 (two-sided), we conducted a sample size calculation using the following assumptions.

**Figure 1:**
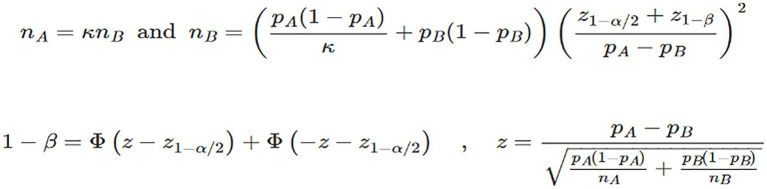
The calculation formula of sample size in this study.

Based on previous studies, we assumed a continued treatment rate of 50% in the intervention group (P1) and 30% in the control group (P2) (1, 7). Considering the potential high dropout rate among the older adult population, we accounted for a 20% dropout rate over the 2-year follow-up period. The calculation resulted in a required sample size of 91 participants per group. Considering a 20% dropout rate, we adjusted the sample size to 114 participants per group, resulting in a total sample size of 228 participants. The sample size is calculated by PASS15.0 software, and the calculation equation is shown in Figure 1.

### 2.11 Statistical analysis

**Figure 2:**
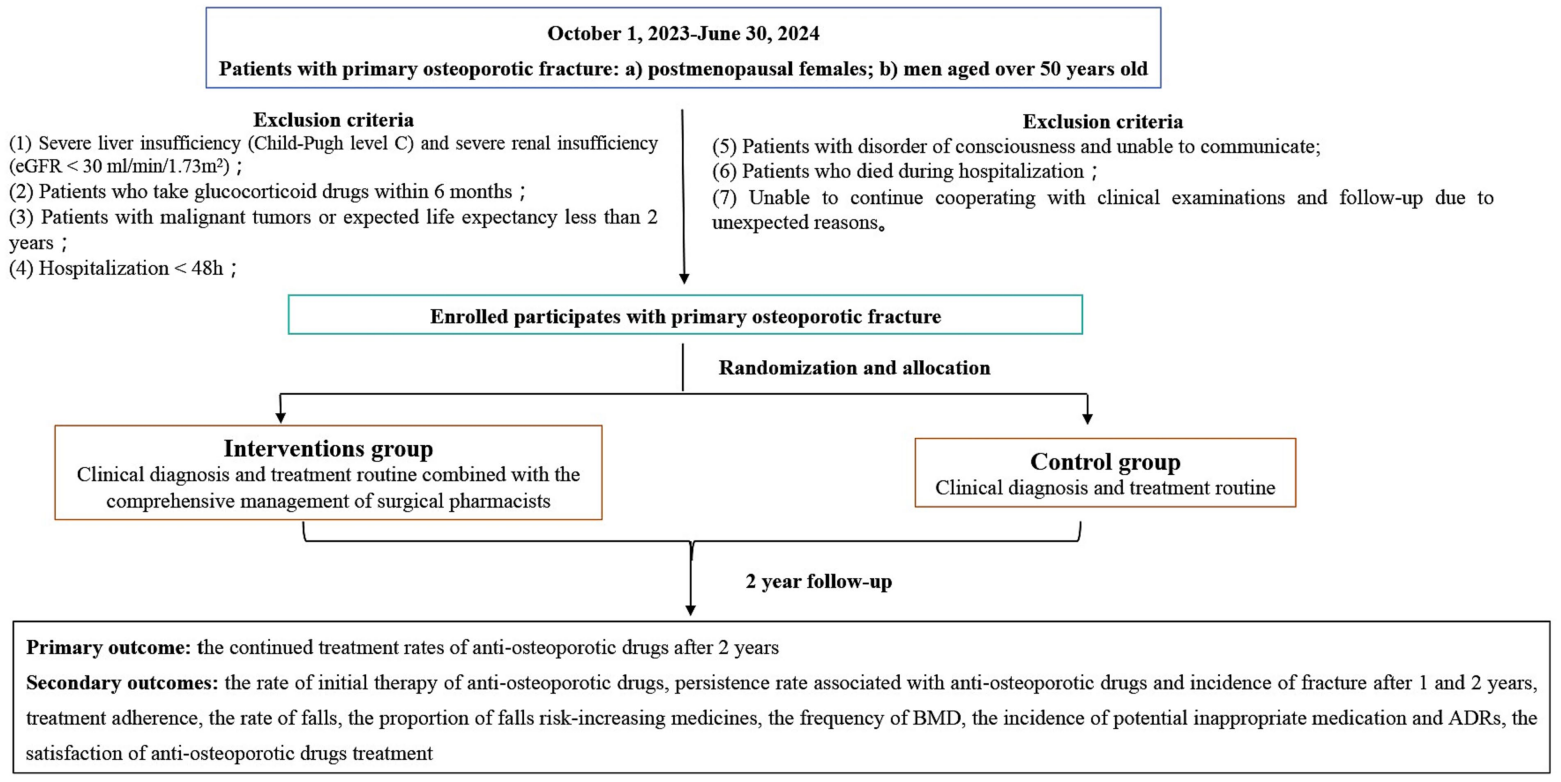
Flow diagram of the study.

**Figure 3:**
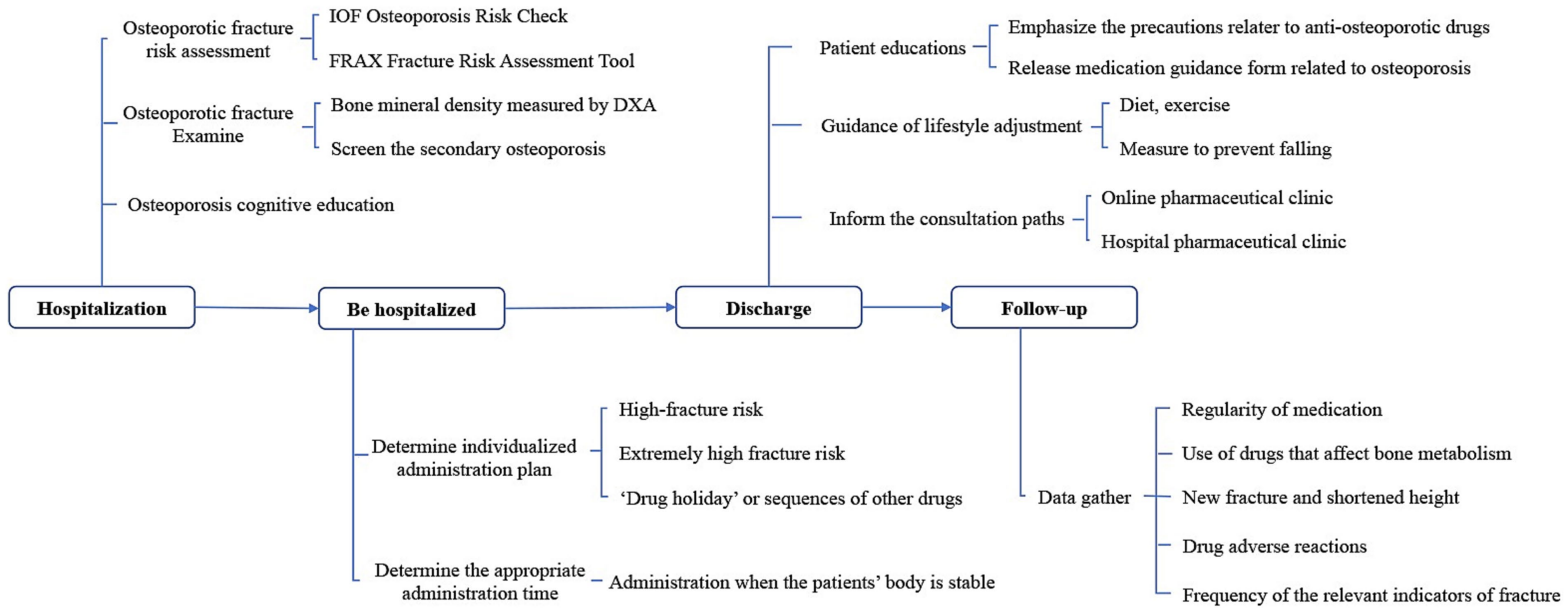
Surgical pharmacist-led intervention pathway of this study.

The statistical analysis will be along with the intention-to-treat (ITT) principles. The ITT population will include patients who meet the criteria, are randomized, and take at least one dose of drugs after being enrolled (Figures 2, 3).

All counting data will be analyzed using the Statistics Package for the Social Science (SPSS) program, version 23.0. Continuous variables are expressed as mean or median (quarterly distance) with standard deviation (mean ± SD), and categorical variables are expressed as frequencies or percentages. The *t*-test will be used to compare continuous data that satisfies normal distribution or the Willcoxon test will be performed for the skewed distribution data. The chi-squared test or Fisher’s exact test will be used to assess the comparison of categorical data in different groups.

For outcomes such as continued treatment rates of anti-osteoporotic drugs, Kaplan–Meier curves will be used to characterize how the probability of an endpoint event changes with survival time, and comparisons between survival curves will be carried out using log-rank tests. We used the Cox proportional hazards model to estimate the hazard ratios and 95% confidence intervals. Missing observations were accounted for using the predictive mean matching (PMM) method. If the missing data are numerical, it will be filled by predictive mean matching; meanwhile, if the missing data are non-numerical, logistic regression and discriminant functions will be used to fill it. The *p*-value threshold is 0.05 (*p* < 0.05), and a p-value below this threshold indicates that the difference is statistically significant.

### 2.12 Ethics issues

Ethical approval was obtained before the start of this protocol from the Committee of Ethics of the First Affiliated Hospital of Shantou University Medical College (approval number: B-2023-194). The study protocol adheres to the principles outlined in the Declaration of Helsinki and complies with all relevant national and institutional guidelines for research involving human participants.

Written informed consent was obtained from all participants. The content form detailed the study objective, procedures, risks, and benefits. Participants were able to withdraw from the study and/or the collection of linked data at any time. Verbal explanations were provided to ensure understanding, and signed forms were securely stored. Participant’s privacy was protected through data anonymization and secure storage. Data were coded with unique identifiers, stored in password-protected databases, and were accessible only to authorized personnel. All staff were required to sign confidentiality agreements, and data sharing adhered to relevant regulations.

## 3 Discussion

This study implements a pharmacist-led intervention to evaluate its effectiveness in the treatment of osteoporotic fractures. Findings from this present study assume that the involvement of pharmacist-led interventions in osteoporotic fracture therapy management will improve anti-osteoporotic drug treatment rates and medication adherence, further decreasing the risk of refracture among Chinese patients with osteoporotic fractures.

To our knowledge, this is the inaugural prospective study to focus on the role of surgical pharmacists in the medication management of osteoporotic fractures in China, with an emphasis on treatment outcomes, prognosis, and medication adherence.

Osteoporotic fracture, a common consequence secondary to osteoporosis (27), results in more socioeconomic burdens than other chronic diseases, such as hypertension, asthma, and rheumatoid arthritis. A previous study has shown that older adults tend to have a higher risk of osteoporosis and further suffer from a secondary fracture, especially in postmenopausal women and men aged above 50 years (6). There is still a gap in osteoporosis treatment, causing a low treatment initiation rate among the elderly, as a result of the lack of awareness of osteoporosis and medication adherence (10).

Several studies have demonstrated that pharmacists should be uniquely positioned in osteoporotic fracture management and contribute to addressing the osteoporosis treatment gap (28). Interventions constructed by pharmacists have exhibited an effect in enhancing osteoporosis management, including medication interviews, patient education and counseling, cooperation with physicians or nurses, and risk assessment of refracture. Pharmacist-led interventions were identified to enhance not only BMD testing and calcium intake but also promote the use of vitamin D supplements and the initiation of osteoporosis treatments (26, 29–32), in addition to medication adherence to anti-osteoporotic drugs. Population screening for patients at a higher risk of fracture is essential for pharmacists to increase osteoporosis treatment rates and reduce the incidence of refractures (33).

A specific medication reconciliation focusing on reduction in falls and subsequent fractures, as well as drug-associated secondary osteoporotic fracture, was recommended by international and Australian osteoporosis management guidelines, if possible (2). Despite a clinical trial demonstrating the effectiveness of medication reconciliation in optimizing medication management for patients with minimal trauma fractures (34), no exact routine of pharmacist-led medication reconciliation has been established. A follow-up interview by pharmacists plays a crucial role in ensuring continuity of care and medication compliance.

Therefore, in this present study, a surgical pharmacist-led intervention was designed for patients with osteoporotic fracture, which contained medication interviews, patient education, counseling, monitoring of potential adverse drug reactions, and up to 10 follow-ups within 24 months. Participants were reminded of tailored precautions for taking medications using telephone interviews, pharmaceutical clinics, and pharmacy practices in e-hospitals, to support adherence to anti-osteoporotic drugs.

There are some limitations to this study. First, only a relatively single center was involved in this study, which means the results may not be representative of all patients with OF. Second, older participants may contribute to a high dropout rate due to physical decline or death. As a result, there is a risk of missing data during follow-up due to these drop-outs. External validations are needed to further investigate the detailed models and roles of surgical pharmacists in the management of therapy and medication compliance of patients with OF.

In conclusion, our results are likely to provide deeper insights into the association between pharmacist-led interventions and medication treatment and adherence to anti-osteoporotic drugs in Chinese elderly patients with osteoporotic fractures. This study will provide valuable data for developing a potential pharmaceutical service model and a longitudinal follow-up visit for patients with OP, aiding in establishing individual intervention and treatment strategies.
